# Association between a mediterranean lifestyle and Type 2 diabetes incidence: a prospective UK biobank study

**DOI:** 10.1186/s12933-023-01999-x

**Published:** 2023-10-04

**Authors:** Javier Maroto-Rodriguez, Rosario Ortolá, Adrián Carballo-Casla, Víctor Iriarte-Campo, Miguel Ángel Salinero-Fort, Fernando Rodríguez-Artalejo, Mercedes Sotos-Prieto

**Affiliations:** 1 Department of Preventive Medicine and Public Health, School of Medicine, Universidad Autónoma de Madrid, Calle del Arzobispo Morcillo 4, Madrid, 28029 Spain; 2 CIBERESP (CIBER of Epidemiology and Public Health), Av. Monforte de Lemos, 3-5. 28029, Madrid, Spain; 3 Aging Research Center, Department of Neurobiology, Care Sciences and Society, Karolinska Institutet & Stockholm University, Stockholm, Sweden; 4 Foundation for Research and Biomedical Innovation of Primary Care of the Community of Madrid (FIIBAP), Av. de la Reina Victoria, 21, 6ª Planta, Madrid, 28003 Spain; 5 Hospital La Paz Institute for Health Research (IdIPAZ), Paseo de la Castellana, 261, Madrid, 28046 Spain; 6 IMDEA-Food Institute. CEI UAM+CSIC, Ctra. de Canto Blanco 8, E. 28049, Madrid, Spain; 7 Department of Environmental Health, Harvard T.H. Chan School of Public Health, 665 Huntington Avenue, Boston, MA 02115 USA

**Keywords:** Diet, Physical activity, Prevention, Adult-onset diabetes, Cohort, Lifestyle medicine

## Abstract

**Background:**

There is mounting evidence that the Mediterranean diet prevents type 2 diabetes, but little is known about the role of Mediterranean lifestyles other than diet and among non-Mediterranean populations. This work aimed to examine the association between a comprehensive Mediterranean-type lifestyle and type 2 diabetes incidence in a British adult population.

**Methods:**

We used data from 112,493 individuals free of cardiovascular disease and type 2 diabetes mellitus, aged 40–69 years, from the UK Biobank cohort, who were followed from 2009 to 2010 to 2021. The Mediterranean lifestyle was assessed through the 25-item MEDLIFE index, which comprises three blocks: (a) “Mediterranean food consumption”, (b) “Mediterranean dietary habits”, (c) “Physical activity, rest, social habits, and conviviality”. Diabetes incidence was obtained from clinical records. Cox proportional-hazards regression models were used to analyze associations and adjusted for the main potential confounders.

**Results:**

After a median follow-up of 9.4 years, 2,724 cases of type 2 diabetes were ascertained. Compared to the first quartile of MEDLIFE adherence, the hazard ratios (95% confidence interval) for increasing quartiles of adherence were 0.90 (0.82–0.99), 0.80 (0.72–0.89) and 0.70 (0.62–0.79) (*p*-trend < 0.001). All three blocks of MEDLIFE were independently associated with lower risk of diabetes.

**Conclusions:**

Higher adherence to the MEDLIFE index was associated with lower risk of type 2 diabetes in the UK Biobank. A Mediterranean-type lifestyle, culturally adapted to non-Mediterranean populations, could help prevent diabetes.

**Supplementary Information:**

The online version contains supplementary material available at 10.1186/s12933-023-01999-x.

## Introduction

Type 2 diabetes mellitus is a metabolic disease, a cardiovascular risk factor and a major cause of disability and death [1]. With an estimated prevalence of 438 million people worldwide in 2019, and an expected increase to 700 million in 2045 [2], diabetes has become a global public health challenge in which lifestyle factors play a key role in its prevention and management [3]. Poor quality diet, sedentary time, low physical activity, smoking, alcohol intake and high BMI are among the main risk factors for diabetes [4]; therefore prevention necessarily requires improved public health strategies that target the environmental factors and lifestyle habits that have a direct or indirect influence on its onset [5].

The Mediterranean lifestyle is a set of behaviors from the people living in the Mediterranean basin, characterized by a plant-based diet with little animal food consumption mainly in form of dairy, fish and white meat, with olive oil as the main fat source, along with other behaviors such us taking short naps (“*siesta*”), adequate night sleep, doing sufficient physical activity and high levels of socialization. Systematic reviews of prospective studies have reported a linear [6] and nonlinear association [7] between the Mediterranean diet (MD) and lower risk of type 2 diabetes in Mediterranean and non-Mediterranean countries. However, no previous study has assessed the association between a Mediterranean-type lifestyle pattern, including diet plus other health behaviors, and type 2 diabetes incidence. In this regard, the MEDLIFE index is a comprehensive score that assesses the adherence to a traditional Mediterranean lifestyle including three main domains: (a) food consumption, (b) dietary habits, and (c) physical activity, rest and social interaction. A higher MEDLIFE score has been previously associated with lower risk of metabolic syndrome and mortality [8], depression [9], frailty [10], cardiovascular disease [11], and chronic disease burden estimated with the GDF-15 [12] in Mediterranean populations. Since the MEDLIFE has already been linked to cardiovascular disease, and diabetes and cardiovascular disease share some risk factors, we hypothesize that higher adherence to the MEDLIFE could be associated with lower diabetes risk, even in a non-Mediterranean country. Moreover, if a Mediterranean lifestyle could be transferred to other populations, it would be a good way to promote healthy behaviors that prevent adverse health outcomes, including diabetes. Although recent studies have used extended lifestyle scores [13, 14] that partly overlap with the MEDLIFE index, the latter is unique because it represents a traditional Mediterranean culture with a comprehensive number of items representing a specific way of living. Thus, we aimed to study the association between the MEDLIFE index and type 2 diabetes incidence in British middle-aged and older adults participating in the UK Biobank study.

## Methods

### Study design and population

The UK Biobank cohort is a multicenter prospective population-based study in the United Kingdom (England, Wales, and Scotland) with around 500,000 participants aged 40–69 years at the time of enrollment. Study methods have been reported elsewhere [15, 16]. Briefly, participants were recruited from 2006 to 2010 in twenty-two assessment centers. At the initial visit, they provided biological samples, completed a touch-screen questionnaire, a computer-assisted interview, and underwent a physical examination. Afterwards, up to five online follow-up 24-hour dietary assessments were completed by some participants (210,965) between 2009 and 2010 (instance 0: April 2009 to September 2010, instance 1: February 2011 to April 2011; instance 2: June 2011 to September 2011; instance 3: October 2011 to December 2011; instance 4: April 2012 to June 2012).

### Study variables

#### Mediterranean lifestyle (MEDLIFE)

Diet at baseline (2009–2012) was assessed via web-based 24-hour dietary assessment (Oxford WebQ) [17] up to five times. In this study we included only those who completed two or more diet assessments, and the average scores were analyzed. From the 126,821 participants with at least two assessments, 48,110 completed two, 42,480 completed three, 30,466 completed four, and 5,765 completed five assessments. Estimation of food groups, nutrients, and energy intake were previously reported [18, 19]. Physical activity was self-reported at baseline at the assessment centers (2006–2010), along with sedentary activities, rest routines, and social habits.

The MEDLIFE index consists of three blocks (20): (a) “Mediterranean food consumption”, with 12 items about food intake (e.g., sweets, vegetables, white meat, fruits, fish); (b) “Mediterranean dietary habits”, with 7 items on habits and practices around meals (e.g., low salt consumption, limit sugar-sweetened beverages); (c) “Physical activity, rest, social habits and conviviality”, with 6 items about physical activity and collective activities (e.g., regular naps, physical activity, adequate sleep, socializing with friends) (Supplementary Table S1). Each item scored 1 point (adherent) or 0 (non-adherent), so the total score ranges from 0 to 25 and higher values represent higher adherence to the Mediterranean lifestyle.

To calculate the MEDLIFE index in the UK Biobank, we had to adjust some items according to previous studies [10, 21]. The modified MEDLIFE index comprised 25 items instead of 29 (Supplementary Table S1) because we could not compute the items for consumption of olive oil and *sofrito* (a traditional sauce with olive oil, tomato, and garlic), for nibbling outside meals, and eating in company, since the UK Biobank did not collect such information.

#### Type 2 diabetes

Prevalent diabetes status (type 1, type 2, gestational, or unclassified) and date of diagnosis at baseline were obtained from self-reports and linkage of clinical records. For incident type 2 diabetes, diagnosis was obtained from self-report, death records, hospital inpatient records and primary care records. Hospital inpatient data were used up to 30th September 2021 for participants in England, up to 31st July 2021 for Scotland, and 31st March 2016 for Wales; primary care data were used up to 31st May 2016 or 31st May 2017 (depending on the data provider) for participants in England, 31st March 2017 for Scotland, and 31st August 2017 for Wales. Participants who died were assigned date of death as a censoring date. Dates of type 2 diabetes incidence correspond to the first reported diagnosis. Cases of type 2 diabetes mellitus were identified with ICD10 code E11.

#### Potential confounders

The following potential confounding factors were chosen based on previous literature on the field. We used self-reported information at baseline on sex, age, ethnicity (white or non-white), educational level (university or not university education), socio-economic status (Townsend deprivation index) and smoking status. Body mass index (BMI) was calculated as weight (kg) divided by squared height (m), both measured at baseline. Prevalent hypertension was defined as use of hypertensive medication or hypertension diagnosis by self-report and linkage of clinical records (confirmed using ICD10 codification). Use of cholesterol-lowering medication was self-reported. Prevalent cardiovascular disease, a criterion of exclusion, was defined as having suffered heart attack, angina, or stroke, by self-report or linkage from medical records. Family history of diabetes was assessed by self-report at baseline and follow-up.

### Statistical analyses

From the initial 126,821 participants with at least two dietary assessments, we excluded those with missing information on more than two MEDLIFE items (*n* = 106) to avoid exposure misclassification, together with those lacking sociodemographic data (*n* = 877), smoking status (*n* = 220), BMI (*n* = 274) cholesterol medication (*n* = 4), information on chronic disease status (diabetes and cardiovascular disease, *n* = 2,508) and family history of diabetes (*n* = 2,930). Participants with prevalent diabetes (*n* = 2,090) or cardiovascular disease (*n* = 5,309) were also excluded from the analyses. Therefore, the analytical sample included 112,493 participants (Supplementary Figure S1).

To assess the association of MEDLIFE with incident type 2 diabetes, we used multivariable Cox models, using age as the underlying timescale, to estimate hazard ratios (HR) and 95% confidence intervals (CI). The MEDLIFE index (exposure) was modelled in quartiles and per 2 points increments, and the first quartile (lowest adherence) was used as reference. *P*-trends were calculated using MEDLIFE quartiles as continuous variable. Three progressively adjusted models were fitted: Model 1, adjusted for sex, age, ethnicity, educational level, deprivation index, and region of assessment center; Model 2, further adjusted for smoking status and energy intake; and Model 3, further adjusted for hypertension, cholesterol medication, BMI (since it is the main risk factor for type 2 diabetes) and family history of diabetes. We replicated the analyses first for each MEDLIFE block adjusting for the remaining blocks, and second for MEDLIFE individual items, adjusting for the MEDLIFE index excluding the corresponding item. We evaluated deviation from linearity with a 3-knots restricted cubic spline Cox regression model, adjusted as in Model 3. Knots were set at percentiles 10, 50 and 90 of the MEDLIFE distribution (points 7, 10 and 13 respectively). Proportional hazards assumption was assessed plotting the survival probability of diabetes over follow-up for the MEDLIFE quartiles; the graphical representation provided no apparent evidence of violation of the assumption.

In sensitivity analyses, we replicated the main analysis: 1) including participants with cardiovascular disease (*N* = 116,832); 2) only in those having at least three diet assessments (*N* = 70,082) to assess possible deviations from usual diet; 3) excluding wine from the MEDLIFE score and adjusting for alcohol consumption to assess the independent association of the MEDLFE with diabetes independently of wine, a characteristic component of the MD with controversial effects on health (24 items); 4) excluding the cases diagnosed in the first two years of follow-up to account for reverse causality (*N* = 111,900); 5) using the Fine-Gray model for competing mortality risks [22]. Likewise, we excluded the rest of the items from MEDLIFE, one item at a time, to examine whether there was a single item driving the main association. Additionally, to assess if the association was modified by some important predictors of diabetes, including sex, age (< 65, ≥ 65 years), deprivation index (≤ the median, > median), and BMI (< 25, 25-29.9, ≥ 30), we stratified the analysis for such variables and also tested potential interactions entering a multiplicative term in the model. We also stratified analyses by health system data source to assess if the differential coverage modified the results.

Analyses were performed using Stata v. 15.1 (Stata-Corp LLC, College Station, Texas). Statistical significance was set at two-sided *p* < 0.05.

## Results

At baseline, participants had a mean (SD) age of 58.7 years and 57.7% were women. Mean (SD) MEDLIFE score was 9.57 (2.60). Participants with lower MEDLIFE score were more likely to be men, smokers, and hypertensives, and to have lower educational level and higher BMI (Table 1). Compared with the analyzed sample, excluded participants were more likely to be men, older, non-white, former smokers, and hypertensives, to have less education and higher BMI, and to take cholesterol-lowering medication (Supplementary Table S2). 335 participants were lost to follow-up.

**Table 1:** Baseline characteristics of the UK Biobank participants with two or more completed dietary assessments, according to quartiles of the MEDLIFE score (*N* = 112,493)

	MEDLIFE				
	Quartile 1 0–7 p	Quartile 2 8–9 p	Quartile 3 10–11 p	Quartile 4 12–22 p	Total
*n*	24,297	32,226	30,713	25,257	112,493
Sex, female, *n* (%)	12,581 (51.78)	18,047 (56.00)	18,153 (59.11)	156,079 (63.66)	64,860 (57.66)
Age, years, mean (SD)	57.44 (7.99)	58.59 (7.88)	59.16 (7.75)	59.52 (7.64)	58.70 (7.85)
Ethnicity, *n* (%)					
White	23,595 (97.11)	31,301 (97.13)	29,758 (96.89)	24,481 (96.93)	109,135 (97.01)
Non-white	702 (2.89)	925 (2.87)	955 (3.11)	776 (3.07)	3,358 (2.99)
Region of assessment					
England	22,098 (90.95)	29,472 (91.45)	28,154 (91.67)	23,273 (92.14)	102,997 (91.56)
Wales	793 (3.26)	1,011 (3.14)	923 (3.01)	715 (2.83)	3,442 (3.06)
Scotland	1,406 (5.79)	1,743 (5.41)	1,636 (5.33)	1,269 (5.02)	6,054 (5.38)
Education					
University education	9,590 (39.47)	14,656 (45.48)	15,508 (50.49)	14,385 (56.95)	54,139 (48.13)
Non-university education	14,707 (60.53)	17,570 (54.52)	15,205 (49.51)	10,872 (43.05)	58,354 (51.87)
Deprivation index, mean (SD)	-1.57 (2.83)	-1.72 (2.78)	-1.75 (2.80)	-1.64 (2.84)	-1.68 (2.81)
Smoking status, *n* (%)					
Never	13,457 (55.39)	18,648 (57.87)	18,393 (59.89)	15,349 (60.77)	65, 847 (58.53)
Former	8,388 (34.52)	11,219 (34.81)	10,676 (34.76)	8,786 (34.79)	39,069 (34.73)
Current	2,452 (10.09)	2,359 (7.32)	1,644 (5.35)	1,122 (4.44)	7,577 (6.74)
Energy intake, kcal/day, mean (SD)	2163 (509)	2079 (495)	2026 (482)	1986 (475)	2062 (494)
Hypertension, *n* (%)	5,699 (23.46)	7,170 (22.25)	6,389 (20.80)	4,809 (19.04)	24,067 (21.39)
Cholesterol-lowering medication, *n* (%)	2,537 (10.44)	3,310 (10.27)	2,916 (9.49)	2,127 (8.42)	10,890 (9.68)
BMI, *n* (%)					
< 25 kg/m^2^	7,705 (31.71)	12,268 (38.07)	13,384 (43.58)	13,343 (52.83)	46,700 (41.51)
25–29.9 kg/m^2^	10,408 (42.84)	13,647 (42.35)	12,657 (41.21)	9,199 (36.42)	45,911 (40.81)
≥ 30 kg/m^2^	6,184 (25.45)	6,311 (19.58)	4,672 (15.21)	2,715 (10.75)	19,882 (17.67)
Family history of diabetes, yes, *n* (%)	5,436 (22.37)	6,902 (21.42)	6,386 (20.79)	4,920 (19.48)	23,644 (21.02)
MEDLIFE index, 0–25 p, mean (SD)	6.11 (1.06)	8.53 (0.50)	10.46 (0.50)	13.14 (1.31)	9.57 (2.60)
Block 1: *Mediterranean food consumption*, 0–12 p, mean (SD)	2.12 (1.08)	3.13 (1.16)	4.05 (1.22)	5.49 (1.43)	3.69 (1.70)
Block 2: *Mediterranean eating habits*, 0–7 p, mean (SD)	2.26 (1.03)	3.05 (1.04)	3.59 (1.05)	4.21 (1.06)	3.29 (1.24)
Block 3: *Physical activity, rest, social habits, and conviviality*; 0–6 p, mean (SD)	1.73 (0.94)	2.34 (1.03)	2.82 (1.08)	3.45 (1.11)	2.59 (1.20)

Abbreviations: *N*, total number of participants; *n*, number of participants included in the category; p, points; SD, standard deviation; BMI, body mass index

**Fig. 1:**
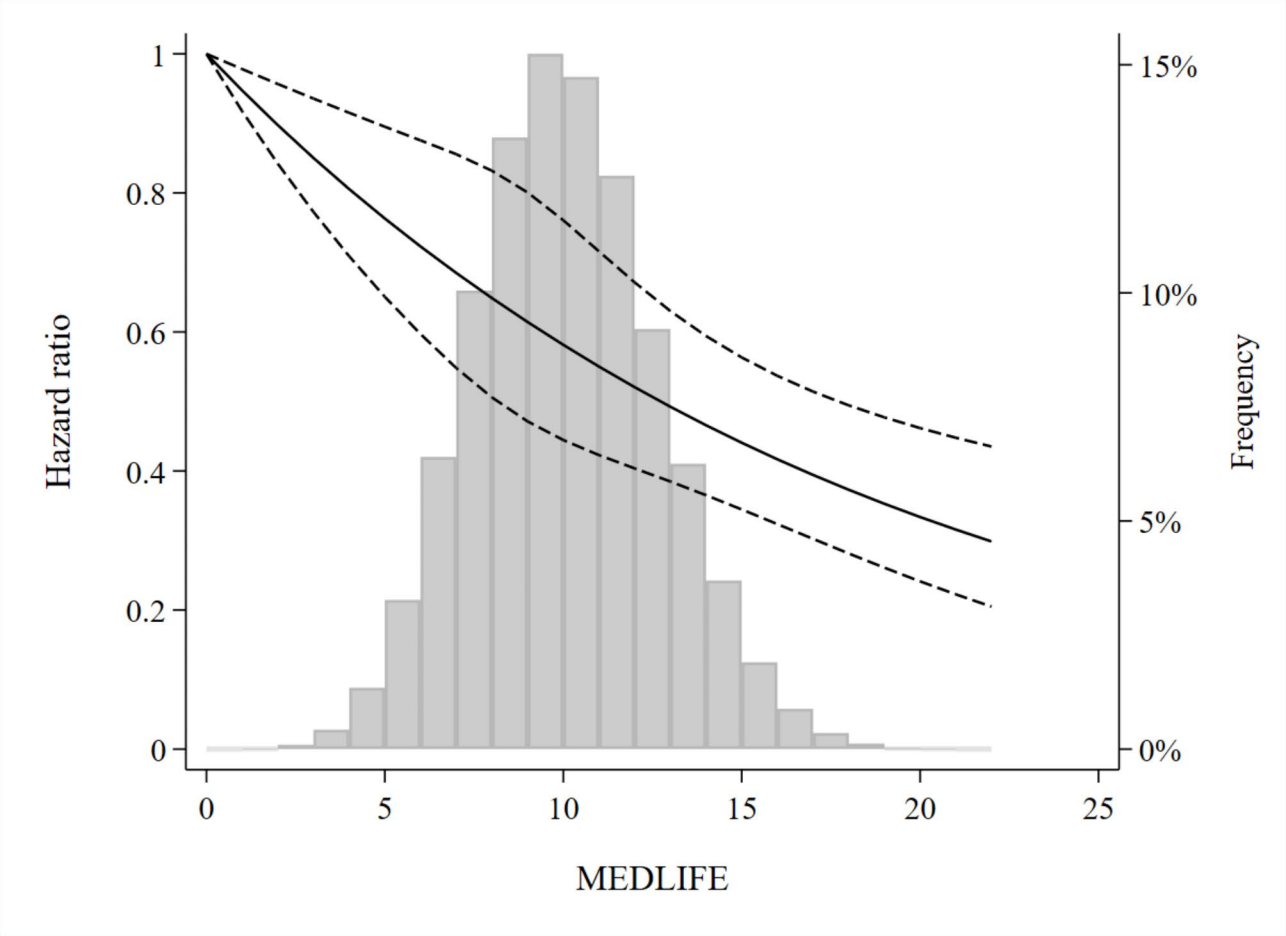
Associations between adherence to the MEDLIFE index and risk of type 2 diabetes in the UK Biobank (*N* = 112,493). Plotted values are hazard ratios (95% confidence intervals) from a restricted cubic spline Cox regression model, adjusted for sex, age, ethnicity, education, deprivation index, region of assessment, smoking status, energy intake, hypertension, cholesterol medication, body mass index, and family history of diabetes. Histogram of frequencies of MEDLIFE score in the background. Abbreviations: *N*, total number of participants

During a median follow-up of 9.41 years (1,041,323.7 person-years), 2,724 incident cases of type 2 diabetes were ascertained (Table 2). Out of these 2,724 cases, 220 were from primary care and 2,341 from hospital inpatient data. We found an inverse dose-response association between the MEDLIFE index and type 2 diabetes. In the most adjusted model, compared to the participants in the first quartile of MEDLIFE, those in the second, third and fourth quartiles had HR of diabetes (95% CI) of 0.90 (0.82–0.99), 0.80 (0.72–0.89), and 0.70 (0.62–0.79), respectively (*p*-trend < 0.001). Per each 2 points increase in MEDLIFE, the HR was 0.90 (0.87–0.92) (*p* < 0.001) (Table 2). In the restricted cubic spline analysis, we observed an almost linear dose-response association (Fig. 1).

**Table 2:** Hazard ratios (95% confidence interval) for the association between MEDLIFE index and risk of type 2 diabetes in the UK Biobank (*N* = 112,493)

	Quartile 1 0–7 p	Quartile 2 8–9 p	Quartile 3 10–11 p	Quartile 4 12–22 p	*p*-trend	Per 2 points increase
*cases/n*	816/24,297	863/32,226	653/30,713	392/25,257		2,724/112,493
Model 1	Ref.	0.80 (0.73–0.88)	0.64 (0.57–0.71)	0.48 (0.42–0.54)	< 0.001	0.81 (0.78–0.83)
Model 2	Ref.	0.81 (0.73–0.99)	0.65 (0.58–0.72)	0.49 (0.43–0.55)	< 0.001	0.81 (0.79–0.84)
Model 3	Ref.	0.90 (0.82–0.99)	0.80 (0.72–0.89)	0.70 (0.62–0.79)	< 0.001	0.90 (0.87–0.92)

Abbreviations: *N*, total number of participants; *n*, number of participants included in each quartile; p, points; Ref., reference

Model 1: Adjusted for sex, age, ethnicity, education, and deprivation index, and region of assessment

Model 2: Adjusted for Model 1 + smoking status, and energy intake

Model 3: Adjusted for Model 2 + hypertension, cholesterol-lowering medication, body mass index, and family history of diabetes

**Fig. 2:**
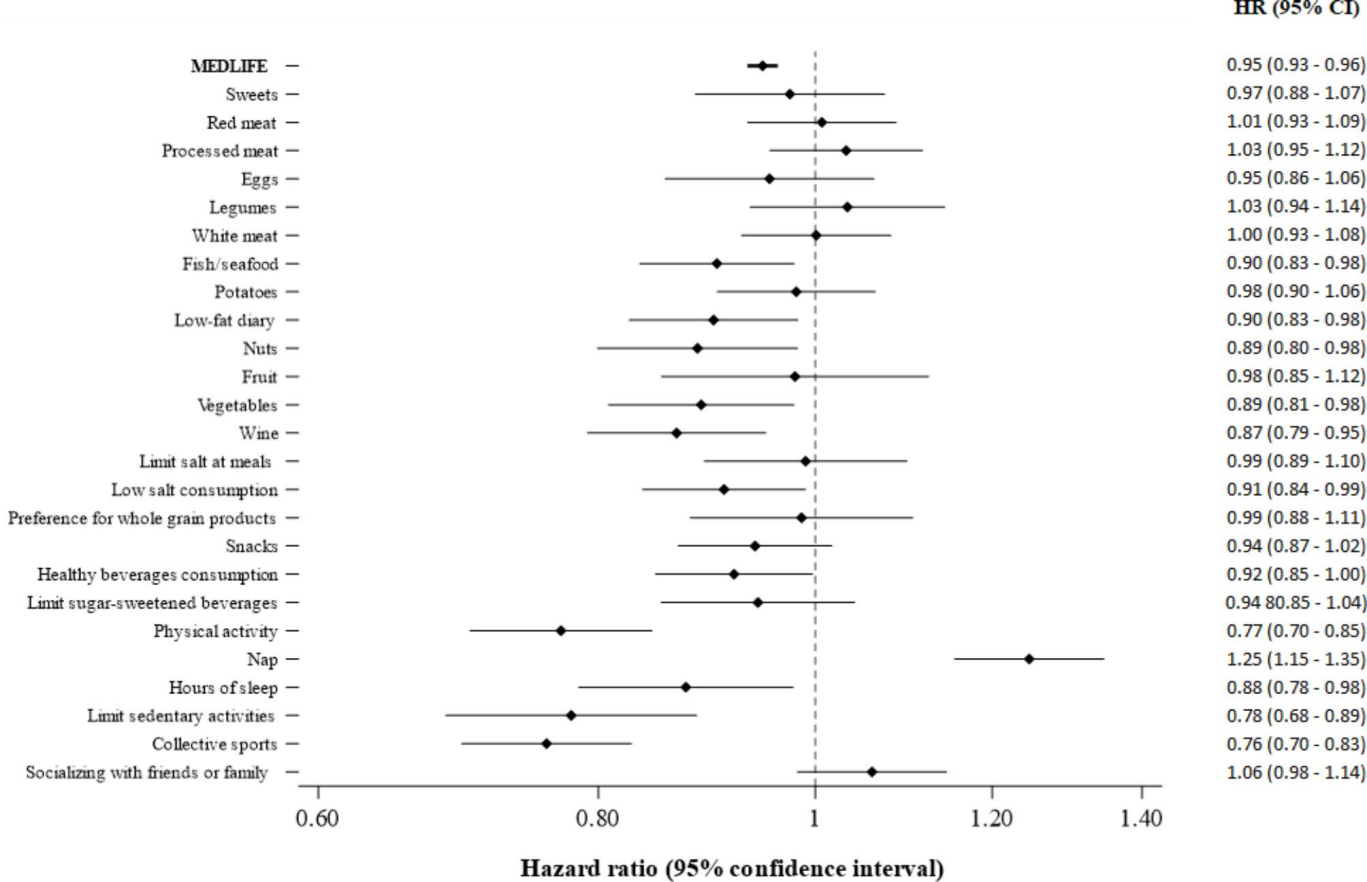
Associations between each MEDLIFE item and risk of type 2 diabetes in the UK Biobank (*N* = 112,493**).** 1 vs. 0 points. Adjusted for sex, age, ethnicity, education, deprivation index, region of assessment, smoking status, energy intake, hypertension, cholesterol-lowering medication, body mass index, and family history of diabetes. Abbreviations: *N*, total number of participants

All MEDLIFE blocks were independently associated with lower type 2 diabetes risk (Table 3). In the most adjusted model, per 2 points increments in the MEDLIFE the HR (95% CI) were 0.93 (0.88–0.97) (*p* = 0.001) for Block 1 (“Mediterranean food consumption”), 0.86 (0.81–0.92) (*p* < 0.001) for Block 2 (“Mediterranean dietary habits”), and 0.88 (0.82–0.93) (*p* < 0.001) for Block 3 (“Physical activity, rest, social habits and conviviality”. Most of the individual components of the MEDLIFE index were associated with diabetes risk in the expected direction (Fig. 2). Furthermore, low-fat dairy, nuts, vegetables, moderate wine consumption, low salt consumption, limiting snacks, doing physical activity, sleeping 6–8 h/day, limiting sedentary activities, and engaging in collective sports were all independently associated with lower risk of diabetes. Conversely, napping usually or sometimes throughout the week was associated with higher risk (Fig. 2).

The results remained very robust in sensitivity analyses: including participants with cardiovascular disease, limiting analyses to participants with three diet assessments or more, excluding wine from the MEDLIFE score, excluding the cases within the first two years of follow-up, and after using a competing risk analysis considering death as a competing event (Supplementary Tables S3-S11), and excluding each item from MEDLIFE (Supplementary Figure S2). In stratified analyses, the association remained in all strata except in participants with a BMI.

**Table 3:** Hazard ratios (95% confidence interval) for the association between MEDLIFE blocks and risk of type 2 diabetes in the UK Biobank (*N* = 112,493**)**

	Per 2 points	*p*-value
**Block 1**: ***Mediterranean food consumption***		
Model 1	0.87 (0.83–0.91)	< 0.001
Model 2	0.87 (0.83–0.91)	< 0.001
Model 3	0.93 (0.88–0.97)	0.001
**Block 2**: ***Mediterranean dietary habits***		
Model 1	0.73 (0.69–0.78)	< 0.001
Model 2	0.74 (0.70–0.79)	< 0.001
Model 3	0.86 (0.81–0.92)	< 0.001
**Block 3**: ***Physical activity, rest, social habits, and conviviality***		
Model 1	0.78 (0.73–0.83)	< 0.001
Model 2	0.78 (0.73–0.83)	< 0.001
Model 3	0.88 (0.82–0.93)	< 0.001

Abbreviations: *N*, total number of participants

Model 1: Adjusted for sex, age, ethnicity, education, and deprivation index, region of assessment, and the remaining blocks

Model 2: Adjusted for Model 1 + smoking status, and energy intake

Model 3: Adjusted for Model 2 + hypertension, cholesterol-lowering medication, body mass index, and family history of diabetes

## Discussion

In this cohort of middle-aged and older adults from the UK, the MEDLIFE score was associated in a dose-response manner with lower risk of type 2 diabetes. Additionally, the three blocks of MEDLIFE were independently associated with lower type 2 diabetes risk.

Our findings remark the importance of not only following a healthy diet or doing physical activity but adhering to an overall healthful lifestyle. Also, this work highlights the possibility to transpose and adapt a Mediterranean-type lifestyle to non-Mediterranean countries; indeed, the fact that in this analysis the MEDLIFE did not include olive oil or *sofrito*, which are characteristic components of the Mediterranean diet, has not precluded observing a beneficial association between a Mediterranean-like lifestyle and diabetes. Notwithstanding this, not considering olive oil may have underestimated the effect of the Mediterranean lifestyle because of the well-known inverse association between olive oil consumption and diabetes [23]. Likewise, when we excluded other items from the MEDLIFE score, the results remained similar; this suggests that the benefits of the Mediterranean-type lifestyle can be achieved even without other traditional characteristics of Mediterranean diet such as wine intake, and that they result mostly from the overall lifestyle pattern rather than from a few of its individual components.

In our study we observed a 30% lower risk of type 2 diabetes for the highest versus lowest quartile of MEDLIFE. These results are in line with those of a meta-analysis of studies assessing different lifestyles in relation to type 2 diabetes [24]. For instance, a set of factors including a healthy BMI, non-smoking status, moderate alcohol consumption, physical activity, high-quality diet, and good sleep pattern was associated with lower risk of diabetes [25]. Adherence to healthful diet and lifestyle recommendations has also been linked to lower diabetes incidence in Australian [26], Swedish [27], and US African-American populations [28]. Furthermore, a prior study in the UK Biobank combining ideal cardiovascular lifestyle and genetic predisposition for type 2 diabetes found both to predict incident diabetes but the effects of a poor lifestyle were comparable across different genetic risk groups [29]. However, most of the previous studies did not assess lifestyle so comprehensively; also, some include intermediate biomarkers of disease as part of the lifestyle but not cultural habits or conviviality.

In this analysis, we have found that the three blocks of MEDLIFE were independently associated with a lower risk of type 2 diabetes. The Block 1 (“Mediterranean food consumption”) represents the MD, and its protective association with diabetes is strongly supported by multiple reviews and meta-analyses of the literature [6, 7]. Many food groups contributed to this association, including vegetables, low-fat dairy, fish & seafood, and nuts. While results for vegetables and low-fat dairy are rather consistent with the literature [30, 31], the existing studies for the last two groups have reported mixed results [32, 33]. Particularly, it seemed that nut consumption was inversely associated with diabetes when BMI, a mediating/modifying variable, was removed from the models.

Regarding Block 2 (“Mediterranean dietary habits”), we had previously found an independent association with the other health outcomes (i.e. metabolic syndrome) [21] in a previous study but not in others (i.e. frailty, pain) [10, 34]. Within this Block, moderate wine consumption, low consumption of salt and low consumption of snacks were independently associated with lower type 2 diabetes incidence. Recent research with the UK Biobank found U-shaped relationships between wine intake and reduced diabetes risk [35, 36]. Other studies have found a direct association between high salt intake and type 2 diabetes [37, 38], but more investigation is needed, given that salt consumption is usually only assessed as a risk factor for hypertension and CVD. However, a link has been reported between high blood pressure and diabetes [39] and both share biological risk factors and pathophysiological mechanisms [40]. On the other hand, snacks are rich in processed ingredients, sugar, and salt, and have been associated with higher risk of diabetes in the context of processed foods in the UK Biobank [41].

Finally, Block 3 (“Physical activity, rest, social habits and conviviality”) was also strongly associated with lower risk of type 2 diabetes, which concurs with previous studies [8, 10, 12, 34, 42]. The association of physical activity and sedentary time with risk of developing diabetes has been previously described [43, 44], also in the UK Biobank [45]. In addition, sleep has often shown a U-shaped relationship with type 2 diabetes, posing short and long sleep a higher risk [46]. Regarding socialization and conviviality, some studies have linked a good social environment to lower risk of diabetes [47], and social integration with lower mortality from type 2 diabetes [48]. This supports the findings for the association between collective sports and diabetes in our analysis since this item combines socialization with physical activity. However, we did not find significant results for socializing with family and friends. It is possible that dichotomizing this item affected our results since we could not account for gradients in socialization.

In our analyses, we found an unexpected direct association between napping and diabetes. In prior MEDLIFE studies, we found that short naps were inversely associated with all-cause mortality [42] and CVD disease [11]. Furthermore, Yamada et al. [49]., found a J-shaped association between short daytime napping and type 2 diabetes and metabolic syndrome, with risk starting to increase at 40 min/day. However, in an umbrella review of studies [50], daytime napping < 40 min was not associated with diabetes risk in young and middle-age adults, but was linked to higher risk for older adults. Two reasons might explain our findings: (1) there was not detailed information about the duration or frequency of naps in the UK Biobank, so we could not account for the complete definition of the original item; (2) napping still carries a stigma in Anglo-Saxon countries [51], and people who take naps might have subclinical conditions or chronic diseases (for instance, they had more prevalence of sleep apnoea (data not shown)); hence reverse causation is possible.

Our findings were robust in several sensitivity analyses. Of note is the significant interaction observed between the MEDLIFE and BMI. The stratified analyses showed significant association for overweight and obese participants only, which showed most of the cases. In our study, the HR (per 2 points increases) went from 0.81 for Model 2 to 0.89 for Model 3 (where BMI and cardiovascular risk factors were included). After excluding BMI from Model 3, the HR went down from 0.90 to 0.83 (0.80–0.86) (data not shown). It is likely that BMI partly mediates the MEDLIFE-type 2 diabetes association, because excess of adiposity is the single most important determinant [52]. Indeed, prior mediation analyses in the UK Biobank showed that higher adherence to the MD resulted in a 14% decreased risk of type 2 diabetes, from which 10% was mediated by overweight [53].

### Underlying mechanisms

The MEDLIFE index was designed to include different lifestyle behaviors − with interrelated biological mechanisms − characterizing the Mediterranean culture and way of living and that may have synergistic effects. Some underlying biological mechanisms of the effect of the MD on type 2 diabetes include: (1) the antioxidant and anti-inflammatory properties of this diet pattern (including vitamins, minerals and polyphenols) that improves HbA1c levels, fasting glucose homeostasis, insulin levels and insulin resistance index (HOMA) [54]. (2) High intake of dietary fiber from multiple plant sources (vegetables, fruits, legumes, whole grains), which increases satiety and reduces insulin resistance, postprandial glucose excursions, inflammation and weight gain [55]. (3) High intake of polyunsaturated fatty acids from nuts and fish, which reduces inflammation biomarkers such as CRP, IL-6 and adiponectin, and have anti-inflammatory effects at endothelium level [56]. Tea and coffee have similar effects on interleukines and TNF, in addition to inhibiting glucose transport and, thus, controlling glucose levels [57].

Physical activity have many benefits on cardiometabolic health: it maintains arterial compliance and function, reduces systemic inflammation biomarkers and reactive oxygen species, enhances antioxidant proteins, reduces mitochondrial fission and improves insulin sensitivity [58]. Poor rest and sleep modify levels of ghrelin, leptin and glucagon-like peptide 1, hormones related to caloric intake and appetite, and insulin sensitivity [59]. In summary, different components of a healthy lifestyle likely have a synergistic effect on systemic inflammation, oxidative stress, glycaemic index, satiety, insulin resistance, and weight maintenance, all of which contribute to the development of type 2 diabetes.

### Strengths and limitations

Strengths of this study include the large number of participants, its prospective design, the long follow-up, the use of a score (MEDLIFE) to assess lifestyle which was previously validated in a Mediterranean population [60] and has already been used in other countries [21, 61], and the high reliability of clinical data, minimizing losses to follow-ups. Nonetheless, some limitations must be acknowledged. First, we have used mostly self-reported data on exposures, and the net effect of measurement errors is difficult to anticipate since they can be both differential and nondifferential. Second, changes in lifestyle over time could not be assessed because of lack of information and the progressively smaller number of participants during the follow-up. Third, despite we have adjusted the analyses for many known potential confounders, some residual confounding might persist due to the observational nature of this study. Fourth, participants were recruited voluntarily, which may introduce some bias; in addition, prior analyses have suggested that participants completing more dietary assessments tended to be older and more educated than the general population of the UK Biobank [62]. Fifth, the coverage of incident diabetes data from primary care is limited; therefore, the incidence of this disease may have been underestimated. To assess the potential for bias, we ran an analysis using only hospital in patient data and another using only primary care data, and both results were very similar. Nevertheless, the UK biobank is able to render generalizable associations in spite of its limitations in terms of representativity [63]. Sixth, the use of at least two diet assessments may not reflect usual diet, but our analysis with three or more dietary assessments produced similar results. Finally, the unavailability of data on four MEDLIFE items, some of them very characteristic of Mediterranean culture such as olive oil, might have underestimated the study associations.

## Conclusions

In this cohort of middle-age and older adults from the United Kingdom, higher adherence to the MEDLIFE index was associated with lower risk of type 2 diabetes, in a dose response manner. A Mediterranean-type lifestyle, culturally adapted to non-Mediterranean populations, could help prevent type 2 diabetes and could be used to promote a healthy lifestyle in public health policies.

### Electronic supplementary material

Below is the link to the electronic supplementary material.

Supplementary Material 1

## Data Availability

The data underlying this article were provided by UK Biobank under a project application.
