# Supplementary material for: Association between a mediterranean lifestyle and Type 2 diabetes incidence: a prospective UK biobank study
Source: Cardiovasc Diabetol. 2023 Oct 4;22:271. doi: 10.1186/s12933-023-01999-x (PMC10552305; doi:10.1186/s12933-023-01999-x)
Supplement: Supplementary file 1 — Supplementary Material 1 [file 12933_2023_1999_MOESM1_ESM.docx]

**Supplementary Table S1.** Description of the Mediterranean Lifestyle (MEDLIFE) index, adapted to the UK Biobank (*N* = 112,493).

| **Index items** | **Components (serving size)** | **Criteria for 1 point** | **% scoring 1 point** |
| --- | --- | --- | --- |
| Block 1: *Mediterranean food consumption* | | |  |
| 1. Sweets | Cookies, chocolate cookies, pastries, donuts, homemade baked goods, store-bought baked goods, muffins (50 g), chocolates (30 g). | ≤ 2 serv/wk | 20.37 |
| 2. Red meat | Beef, pork, lamb (125 g). | ≤ 2 serv/wk | 53.63 |
| 3. Processed meat | Sausage, soft spicy sausage, bacon, cured ham, cooked ham, hamburger, liver, organ meats, pâté (42.5 g). | ≤ 1 serv/wk | 44.56 |
| 4. Eggs | Eggs (1 unit = 65 g). | ≥ 2 to ≤ 4 serv/wk | 14.45 |
| 5. Legumes | Lentils, beans, chickpeas, peas (150 g cooked). | ≥ 2 serv/wk | 16.96 |
| 6. White meat | Chicken/turkey with skin, chicken/turkey without skin, rabbit (125 g). | ≤ 2 serv/wk | 60.39 |
| 7. Fish/seafood | White fish, fatty fish, codfish, salted or smoked fish, shrimp, octopus, calamari, oysters and shellfish (125 g). | ≥ 2 serv/wk | 38.63 |
| 8. Potatoes | Baked or boiled potatoes (150 g). | ≤ 3 serv/wk | 37.97 |
| 9. Low-fat dairy products | Skim milk, low-fat milk (200 ml), low fat yogurt (125 g), fresh soft cheese (50 g). | 2 serv/d | 28.56 |
| 10. Nuts | Almonds, peanuts, hazelnuts, walnuts (30 g). | ≥ 3 serv/wk | 20.26 |
| 11. Sofrito | Sauce of olive oil with onion, pepper, other vegetables (250 g) and tomato (150 g). | > 2/4 ingredients above the median | --- |
| 12. Fruit | Orange, banana, apple, pear, kiwi, mango, avocado, peach, apricot, nectarine, clementine, strawberry, cherries, plums, figs, grapes, watermelon, melon (160 g), dates and dried fruits (30 g). | ≥ 3 serv/d | 9.29 |
| 13. Vegetables | Spinach, cauliflower, broccoli, lettuce, carrot, squash, green beans, eggplant, zucchini, cucumber, pepper, asparagus, gazpacho, garden salad, tomato, other vegetables (236.59 g for green leafy, half for others) *(excluding potatoes).* | ≥ 2 serv/d | 24.09 |
| 14. Olive oil | Olive oil (1 Tbsp = 13.5 g) | ≥ 3 serv/d | --- |
| Block 2: *Mediterranean* d*ietary habits* | | | |
| 15. Wine | Red/white wine (1 glass, 100 ml). | Women: > 0 to ≤ 1 serv/d  Men: > 0 to ≤ 2 serv/d | 26.02 |
| 16. Limit salt at meals | Do you add salt to your food? (*After cooking*). | “Never” or “Sometimes” | 86.81 |
| 17. Low salt consumption | Low sodium-to-potassium ratio and low sodium consumption. | Na/K ratio ≤ 0.57  Na ≤ 2.000 mg/d | 48.30 |
| 18. Preference for whole grain products | Fiber from whole grain cereals. | > 6 g/d fiber from cereals | 15.28 |
| 19. Snacks | Potato chips, popcorn, or other chips (50 g). | ≤ 1 serv/wk | 62.35 |
| 20. Limit snacking between meals | Do you tend to snack in between meals or before going to bed? | No | --- |
| 21. Healthy beverages consumption | Coffee, decaffeinated coffee (1 cup = 50 ml) or tea (1 cup = 250 ml). | ≥ 1 to ≤ 4 serv/d | 71.15 |
| 22. Limited consumption of sugar-sweetened beverages | Sugar-sweetened beverages + juice (150 ml). | < 1/wk | 19.02 |
| Block 3: *Physical activity, rest social habits and conviviality* | | | |
| 20. Physical activity | Brisk walking, jogging, running, climbing stairs, bicycling, stationary cycling, swimming, dance, aerobic exercise, martial arts, gymnastics, gardening, tennis, soccer, skiing, ice skating, team sports, and other physical activities or sports. | ≥ 150 min of moderate, 75 min vigorous, or an equivalent combination | 30.95 |
| 24. Nap | Napping throughout the week. | “Usually” or “Sometimes” if night-time sleep is adequate | 33.31 |
| 25. Hours of sleep | Sleeping throughout the week. | 6-8 h/d | 90.16 |
| 26. Limit sedentary activities | Watching TV, using internet or driving. | ≤ 2 h/d | 20.62 |
| 27. Eating in company | Do you have lunch or dinner with friends, family, or others? | Yes | --- |
| 28. Collective sports | Playing soccer, tennis, squash, basketball, or other team sports, running in group, jogging; etc. | ≥ 1 h/wk | 38.18 |
| 29. Socializing with friends or family | Frequency on what you see family (different from whom you live with) or friends (home, church, bars…). | ≥ 3 times/wk | 45.75 |

Abbreviations: *N*, total number of participants; serv, serving; d, day, wk, week; Tbsp, tablespoons; TV, television.

**Supplementary Table S2**. Baseline characteristics of the UK Biobank participants by inclusion status in the analytical sample.

|  | **Included** | **Excluded** |
| --- | --- | --- |
| *N* | 112,493 | 14,328 |
| Sex, female, *n* (%) | 64,860 (57.66) | 5,841 (40.77) |
| Age, years, mean (SD) | 58.70 (7.85) | 61.79 (7.37) |
| Ethnicity, *n* (%) |  |  |
| White | 109,135 (97.01) | 13,195 (92.09) |
| Non-white | 3,358 (2.99) | 703 (4.91) |
| Region of assessment |  |  |
| England | 102,997 (91.56) | 13,149 (91.77) |
| Wales | 3,442 (3.06) | 528 (3.69) |
| Scotland | 6,054 (5.38) | 651 (4.54) |
| Education |  |  |
| University education | 54,139 (48.13) | 5,319 (37.12) |
| Non-university education | 58,354 (51.87) | 8,610 (60.09) |
| Deprivation index, mean (SD) | -1.68 (2.81) | -1.27 (3.03) |
| Smoking status, *n* (%) |  |  |
| Never | 65, 847 (58.53) | 6,533 (45.60) |
| Former | 39,069 (34.73) | 6,297 (43.95) |
| Current | 7,577 (6.74) | 1,223 (8.54) |
| Energy intake, kcal/day, mean (SD) | 2062 (494) | 2061 (518) |
| Hypertension, *n* (%) | 24,067 (21.39) | 7,676 (53.57) |
| Cholesterol medication, *n* (%) | 10,890 (9.68) | 7,794 (54.40) |
| BMI, *n* (%) |  |  |
| < 25 | 46,700 (41.51) | 3,352 (23.39) |
| 25 – 29.9 | 45,911 (40.81) | 5,726 (39.96) |
| ≥ 30 | 19,882 (17.67) | 4,962 (34.63) |
| Family history of diabetes, yes, *n (%)* | 23,644 (21.02) | 3,758 (26.23) |
| MEDLIFE index, 0-25 p, mean (SD) | 9.56 (2.59) | 9.13 (2.62) |
| Block 1: Mediterranean food consumption, 0-12 p, mean (SD) | 3.69 (1.70) | 3.58 (1.66) |
| Block 2: Mediterranean eating habits, 0-7 p, mean (SD) | 3.29 (1.24) | 3.17 (1.26) |
| Block 3: Physical activity, rest, social habits, and conviviality; 0-6 p, mean (SD) | 2.58 (1.20) | 2.40 (1.21) |

Abbreviations: *N*, total number of participants; p, points; SD standard deviation; BMI, body mass index.

**Supplementary Table S3.** Hazard ratios (95% confidence interval) for the association between MEDLIFE index and risk of type 2 diabetes in the UK Biobank, including participants with cardiovascular disease (*N* = 116,832).

|  | Quartile 1  0 – 7 p | Quartile 2  8 – 9 p | Quartile 3  10 – 11 p | Quartile 4  12 – 22 p | *p*-trend | Per 2 points |
| --- | --- | --- | --- | --- | --- | --- |
| *cases/n* | 934/25,330 | 968/33,520 | 736/31,866 | 444/26,116 |  | 3,082/116,832 |
| Model 1 | Ref. | 0.79 (0.72 – 0.86) | 0.63 (0.57 – 0.70) | 0.48 (0.43 – 0.54) | < 0.001 | 0.81 (0.79 – 0.83) |
| Model 2 | Ref. | 0.80 (0.73 – 0.87) | 0.64 (0.58 – 0.71) | 0.49 (0.44 – 0.55) | < 0.001 | 0.81 (0.79 – 0.84) |
| Model 3 | Ref. | 0.89 (0.81 – 0.98) | 0.80 (0.72 – 0.88) | 0.70 (0.62 – 0.78) | < 0.001 | 0.90 (0.87 – 0.92) |

Abbreviations: *N*, total number of participants; *n*, number of participants included in each quartile; p, points; Ref., reference.

Model 1: Adjusted for sex, age, ethnicity, education, and deprivation index, and region of assessment.

Model 2: Adjusted for Model 1 + smoking status, and energy intake.

Model 3: Adjusted for Model 2 + hypertension, cholesterol-lowering medication, body mass index, family history of diabetes, cardiovascular disease.

**Supplementary Table S4.** Hazard ratios (95% confidence interval) for the association between MEDLIFE index and risk of type 2 diabetes in the UK Biobank cohort, in participants who completed three or more diet assessments (*N* = 70,082).

|  | Quartile 1  1 – 7 p | Quartile 2  8 – 9 p | Quartile 3  10 – 11 p | Quartile 4  12 – 22 p | *p*-trend | Per 2 points |
| --- | --- | --- | --- | --- | --- | --- |
| *cases/n* | 518/15,868 | 511/20,125 | 395 /18,730 | 225/15,359 |  | 1,649/70,082 |
| Model 1 | Ref. | 0.79 (0.70 – 0.89) | 0.66 (0.58 – 0.75) | 0.47 (0.40 – 0.56) | < 0.001 | 0.81 (0.78 – 0.84) |
| Model 2 | Ref. | 0.80 (0.71 – 0.90) | 0.68 (0.59 – 0.77) | 0.49 (0.42 – 0.57) | < 0.001 | 0.82 (0.78 – 0.85) |
| Model 3 | Ref. | 0.90 (0.80 – 1.02) | 0.86 (0.75 – 0.98) | 0.73 (0.62 – 0.85) | < 0.001 | 0.91 (0.88 – 0.95) |

Abbreviations: *N*, total number of participants; *n*, number of participants included in each quartile; p, points; Ref., reference.

Model 1: Adjusted for sex, age, ethnicity, education, and deprivation index, and region of assessment.

Model 2: Adjusted for Model 1 + smoking status, and energy intake.

Model 3: Adjusted for Model 2 + hypertension, cholesterol-lowering medication, body mass index, family history of diabetes, and cardiovascular disease.

**Supplementary Table S5.** Hazard ratios (95% confidence interval) for the association between MEDLIFE index and risk of type 2 diabetes in the UK Biobank, excluding wine from the MEDLIFE (*N* = 112,493).

|  | Quartile 1  0 – 7 p | Quartile 2  8 – 9 p | Quartile 3  10 – 11 p | Quartile 4  12 – 21 p | *p*-trend | Per 2 points |
| --- | --- | --- | --- | --- | --- | --- |
| *cases/n* | 903/27,420 | 874/33,791 | 614/29,461 | 333/21,821 |  | 2,724/112,493 |
| Model 1 | Ref. | 0.80 (0.73 – 0.87) | 0.65 (0.58 – 0.72) | 0.49 (0.43 – 0.56) | < 0.001 | 0.81 (0.79 – 0.84) |
| Model 2 | Ref. | 0.80 (0.73 – 0.88) | 0.65 (0.59 – 0.73) | 0.50 (0.44 – 0.57) | < 0.001 | 0.82 (0.79 – 0.84) |
| Model 3 | Ref. | 0.90 (0.82 – 0.99) | 0.81 (0.73 – 0.90) | 0.70 (0.62 – 0.80) | < 0.001 | 0.90 (0.87 – 0.93) |

Abbreviations: *N*, total number of participants; *n*, number of participants included in each quartile; p, points; Ref., reference.

Model 1: Adjusted for sex, age, ethnicity, education, and deprivation index, region of assessment, and wine consumption.

Model 2: Adjusted for Model 1 + smoking status, and energy intake.

Model 3: Adjusted for Model 2 + hypertension, cholesterol-lowering medication, body mass index, and family history of diabetes.

**Supplementary Table S6.** Hazard ratios (95% confidence interval) for the association between MEDLIFE index and risk of type 2 diabetes in the UK Biobank, excluding cases of type 2 diabetes within first two years of follow-up (*N* = 111,900).

|  | Quartile 1  0 – 7 p | Quartile 2  8 – 9 p | Quartile 3  10 – 11 p | Quartile 4  12 – 22 p | *p*-trend | Per 2 points |
| --- | --- | --- | --- | --- | --- | --- |
| *cases/n* | 642/24,123 | 687/32,050 | 516/30,576 | 286/25,151 |  | 2,131/111,900 |
| Model 1 | Ref. | 0.79 (0.71 – 0.88) | 0.62 (0.55 – 0.70) | 0.43 (0.37 – 0.49) | < 0.001 | 0.79 (0.76 – 0.81) |
| Model 2 | Ref. | 0.80 (0.72 – 0.90) | 0.63 (0.56 – 0.71) | 0.44 (0.38 – 0.51) | < 0.001 | 0.79 (0.76 – 0.82) |
| Model 3 | Ref. | 0.89 (0.80 – 1.00) | 0.78 (0.69 – 0.88) | 0.62 (0.54 – 0.71) | < 0.001 | 0.87 (0.84 – 0.90) |

Abbreviations: *N*, total number of participants; *n*, number of participants included in each quartile; p, points; Ref., reference.

Model 1: Adjusted for sex, age, ethnicity, education, and deprivation index, and region of assessment.

Model 2: Adjusted for Model 1 + smoking status, and energy intake.

Model 3: Adjusted for Model 2 + hypertension, cholesterol-lowering medication, body mass index, and family history of diabetes.

**Supplementary Table S7.** Hazard ratios (95% confidence interval) for the association between MEDLIFE index and risk of type 2 diabetes in the UK Biobank, from subdistribution hazard models considering death as competing event (*N* = 112,493).

|  | Quartile 1  0 – 7 p | Quartile 2  8 – 9 p | Quartile 3  10 – 11 p | Quartile 4  12 – 22 p | *p*-trend | Per 2 points increase |
| --- | --- | --- | --- | --- | --- | --- |
| *cases/n* | 816/24,297 | 863/32,226 | 653/30,713 | 392/25,257 |  | 2,724/112,493 |
| Model 1 | Ref. | 0.76 (0.69 – 0.83) | 0.61 (0.55 – 0.68) | 0.47 (0.40 – 0.54) | < 0.001 | 0.81 (0.79 – 0.83) |
| Model 2 | Ref. | 0.76 (0.70 – 0.83) | 0.62 (0.55 – 0.69) | 0.47 (0.41 – 0.55) | < 0.001 | 0.81 (0.79 – 0.84) |
| Model 3 | Ref. | 0.86 (0.79 – 0.94) | 0.78 (0.70 – 0.87) | 0.68 (0.59 – 0.79) | < 0.001 | 0.90 (0.87 – 0.93) |

Abbreviations: *N*, total number of participants; *n*, number of participants included in each quartile; p, points; Ref., reference.

Model 1: Adjusted for sex, age, ethnicity, education, and deprivation index, and region of assessment.

Model 2: Adjusted for Model 1 + smoking status, and energy intake.

Model 3: Adjusted for Model 2 + hypertension, cholesterol-lowering medication, body mass index, and family history of diabetes.

**Supplementary Table S8.** Hazard ratios (95% confidence interval) for the association between MEDLIFE index blocks and risk of type 2 diabetes in the UK Biobank, including participants with cardiovascular disease (*N* = 116,832).

|  | Per 2 points | *p*-value |
| --- | --- | --- |
| **Block 1: *Mediterranean food consumption*** | | |
| Model 1 | 0.88 (0.84 – 0.92) | < 0.001 |
| Model 2 | 0.88 (0.84 – 0.92) | < 0.001 |
| Model 3 | 0.93 (0.89 – 0.98) | 0.003 |
| **Block 2: *Mediterranean dietary habits*** | | |
| Model 1 | 0.73 (0.69 – 0.77) | < 0.001 |
| Model 2 | 0.74 (0.69 – 0.78) | < 0.001 |
| Model 3 | 0.85 (0.80 – 0.91) | < 0.001 |
| **Block 3: *Physical activity, rest, social habits, and conviviality*** | | |
| Model 1 | 0.78 (0.78 – 0.83) | < 0.001 |
| Model 2 | 0.79 (0.74 – 0.84) | < 0.001 |
| Model 3 | 0.88 (0.83 – 0.94) | < 0.001 |

Abbreviations: *N*, total number of participants.

Model 1: Adjusted for sex, age, ethnicity, education, and deprivation index, region of assessment, and the remaining blocks.

Model 2: Adjusted for Model 1 + smoking status, and energy intake.

Model 3: Adjusted for Model 2 + hypertension, cholesterol-lowering medication, body mass index, and family history of diabetes.

**Supplementary Table S9.** Hazard ratios (95% confidence interval) for the association between MEDLIFE index blocks and risk of type 2 diabetes in the UK Biobank, in participants who completed three or more diet assessments (*N* = 70,082).

|  | Per 2 points | *p*-value |
| --- | --- | --- |
| **Block 1: *Mediterranean food consumption*** | | |
| Model 1 | 0.90 (0.85 – 0.95) | < 0.001 |
| Model 2 | 0.89 (0.84 – 0.95) | < 0.001 |
| Model 3 | 0.96 (0.90 – 1.02) | 0.193 |
| **Block 2: *Mediterranean dietary habits*** | | |
| Model 1 | 0.68 (0.63 – 0.74) | < 0.001 |
| Model 2 | 0.70 (0.65 – 0.76) | < 0.001 |
| Model 3 | 0.83 (0.77 – 0.90) | < 0.001 |
| **Block 3: *Physical activity, rest, social habits, and conviviality*** | | |
| Model 1 | 0.80 (0.74 – 0.87) | < 0.001 |
| Model 2 | 0.80 (0.74 – 0.87) | < 0.001 |
| Model 3 | 0.91 (0.84 – 0.99) | 0.023 |

Abbreviations: *N*, total number of participants.

Model 1: Adjusted for sex, age, ethnicity, education, and deprivation index, region of assessment, and the remaining blocks.

Model 2: Adjusted for Model 1 + smoking status, and energy intake.

Model 3: Adjusted for Model 2 + hypertension, cholesterol-lowering medication, , body mass index, and family history of diabetes

**Supplementary Table S10.** Hazard ratios (95% confidence interval) for the association between MEDLIFE index blocks and risk of type 2 diabetes in the UK Biobank, excluding wine from the MEDLIFE (*N* = 112,493).

|  | Per 2 points | *p*-value |
| --- | --- | --- |
| **Block 1: *Mediterranean food consumption*** | | |
| Model 1 | 0.85 (0.81 – 0.90) | < 0.001 |
| Model 2 | 0.85 (0.81 – 0.90) | < 0.001 |
| Model 3 | 0.91 (0.86 – 0.96) | < 0.001 |
| **Block 2: *Mediterranean dietary habits*** | | |
| Model 1 | 0.70 (0.66 – 0.75) | < 0.001 |
| Model 2 | 0.71 (0.67 – 0.77) | < 0.001 |
| Model 3 | 0.83 (0.77 – 0.89) | < 0.001 |
| **Block 3: *Physical activity, rest, social habits, and conviviality*** | | |
| Model 1 | 0.77 (0.72 – 0.82) | < 0.001 |
| Model 2 | 0.77 (0.72 – 0.82) | < 0.001 |
| Model 3 | 0.85 (0.79 – 0.91) | < 0.001 |

Abbreviations: *N*, total number of participants.

Model 1: Adjusted for sex, age, ethnicity, education, and deprivation index, region of assessment, wine consumption, and the remaining blocks.

Model 2: Adjusted for Model 1 + smoking status, and energy intake.

Model 3: Adjusted for Model 2 + hypertension, cholesterol-lowering medication, body mass index, and family history of diabetes.

**Supplementary Table S11.** Hazard ratios (95% confidence interval) for the association between MEDLIFE index blocks and risk of type 2 diabetes in the UK Biobank, excluding cases of type 2 diabetes within first two years of follow-up (*N* = 111,900).

|  | Per 2 points | *p*-value |
| --- | --- | --- |
| **Block 1: *Mediterranean food consumption*** | | |
| Model 1 | 0.85 (0.81 – 0.90) | < 0.001 |
| Model 2 | 0.85 (0.81 – 0.90) | < 0.001 |
| Model 3 | 0.91 (0.86 – 0.96) | < 0.001 |
| **Block 2: *Mediterranean dietary habits*** | | |
| Model 1 | 0.70 (0.65 – 0.75) | < 0.001 |
| Model 2 | 0.71 (0.66 – 0.76) | < 0.001 |
| Model 3 | 0.82 (0.76 – 0.88) | < 0.001 |
| **Block 3: *Physical activity, rest, social habits, and conviviality*** | | |
| Model 1 | 0.78 (0.72 – 0.84) | < 0.001 |
| Model 2 | 0.78 (0.73 – 0.84) | < 0.001 |
| Model 3 | 0.87 (0.81 – 0.94) | < 0.001 |

Abbreviations: *N*, total number of participants.

Model 1: Adjusted for sex, age, ethnicity, education, and deprivation index, region of assessment, wine consumption, and the remaining blocks.

Model 2: Adjusted for Model 1 + smoking status, and energy intake.

Model 3: Adjusted for Model 2 + hypertension, cholesterol-lowering medication, body mass index, and family history of diabetes.

**Supplementary Table S12.** Hazard ratios (95% confidence interval) for the association between MEDLIFE index and risk of type 2 diabetes in the UK Biobank by different subgroups (*N* = 112,493**)**.

|  | cases/*n* | Per 2 points | *p*-value | Interaction | *p* for interaction |
| --- | --- | --- | --- | --- | --- |
| **Sex** |  |  |  |  |  |
| Men | 1,546/46,087 | 0.88 (0.85 – 0.92) | < 0.001 | 0.97 (0.91 – 1.03) | 0.327 |
| Women | 1,178/63,682 | 0.92 (0.88 – 0.96) | < 0.001 |  |  |
| **Age** |  |  |  |  |  |
| < 65 years | 1,781/84,702 | 0.90 (0.87 – 0.94) | < 0.001 | 1.00 (1.00 – 1.01) | 0.025 |
| ≥ 65 years | 943/27,791 | 0.89 (0.84 – 0.94) | < 0.001 |  |  |
| **Deprivation index** |  |  |  |  |  |
| ≤ -2.38728 | 1,212/56,247 | 0.91 (0.87 – 0.95) | < 0.001 | 0.99 (0.9 – 1.00) | 0.221 |
| > -2.38728 | 1,512/56,246 | 0.89 (0.85 – 0.93) | < 0.001 |  |  |
| **BMI** |  |  |  |  |  |
| < 25 kg/m^2^ | 315/46,700 | 0.97 (0.88 – 1.05) | 0.432 | 0.95 (0.91 – 0.99) | 0.013 |
| 25 – 29.9 kg/m^2^ | 1,027/45,911 | 0.90 (0.86 – 0.95) | < 0.001 |  |  |
| ≥ 30 kg/m^2^ | 1,382/19,882 | 0.89 (0.85 – 0.93) | < 0.001 |  |  |

Abbreviations: *N*, total number of participants; *n*, number of participants included in each strata; BMI.; body mass index.

Adjusted for sex, age, ethnicity, education, and deprivation index, region of assessment, smoking status, energy intake, hypertension, cholesterol-lowering medication, body mass index, and family history of diabetes.

**Supplementary Table S13.** Hazard ratios (95% confidence interval) for the association between MEDLIFE index and risk of type 2 diabetes in the UK Biobank by health system data source.

|  | Quartile 1  0 – 7 p | Quartile 2  8 – 9 p | Quartile 3  10 – 11 p | Quartile 4  12 – 22 p | *p*-trend | Per 2 points |
| --- | --- | --- | --- | --- | --- | --- |
| **From primary care** | |  |  |  |  |  |
| *cases/n* | 71/23,552 | 61/31,424 | 49/30,109 | 39/24,903 |  | 220/109,988 |
| Model 1 | Ref. | 0.68 (0.48 – 0.96) | 0.59 (0.41 – 0.85) | 0.59 (0.40 – 0.88) | 0.004 | 0.82 (0.74 – 0.92) |
| Model 2 | Ref. | 0.67 (0.47 – 0.94) | 0.57 (0.39 – 0.82) | 0.57 (0.38 – 0.85) | 0.003 | 0.81 (0.73 – 0.91) |
| Model 3 | Ref. | 0.76 (0.54 – 1.07) | 0.71 (0.49 – 1.03) | 0.85 (0.57 – 1.27) | 0.265 | 0.91 (0.81 – 1.02) |
| **From hospital inpatient** | |  |  |  |  |  |
| *cases/n* | 703/24,184 | 749/32,112 | 564/30,624 | 325/25,189 |  | 2,341/112,109 |
| Model 1 | Ref. | 0.80 (0.72 – 0.89) | 0.63 (0.57 – 0.71) | 0.45 (0.40 – 0.52) | < 0.001 | 0.80 (0.77 – 0.83) |
| Model 2 | Ref. | 0.81 (0.73 – 0.90) | 0.65 (0.58 – 0.72) | 0.47 (0.41 – 0.53) | < 0.001 | 0.81 (0.78 – 0.83) |
| Model 3 | Ref. | 0.90 (0.81 – 1.00) | 0.80 (0.72 – 0.90) | 0.66 (0.58 – 0.76) | < 0.001 | 0.89 (0.86 – 0.92) |

Abbreviations: *n*, number of participants included in each quartile; p, points; Ref., reference.

Model 1: Adjusted for sex, age, ethnicity, education, and deprivation index, and region of assessment.

Model 2: Adjusted for Model 1 + smoking status, and energy intake.

Model 3: Adjusted for Model 2 + hypertension, cholesterol-lowering medication, body mass index, and family history of diabetes.

**Supplementary Figure S1.** Flow diagram of included participants from the UK Biobank.


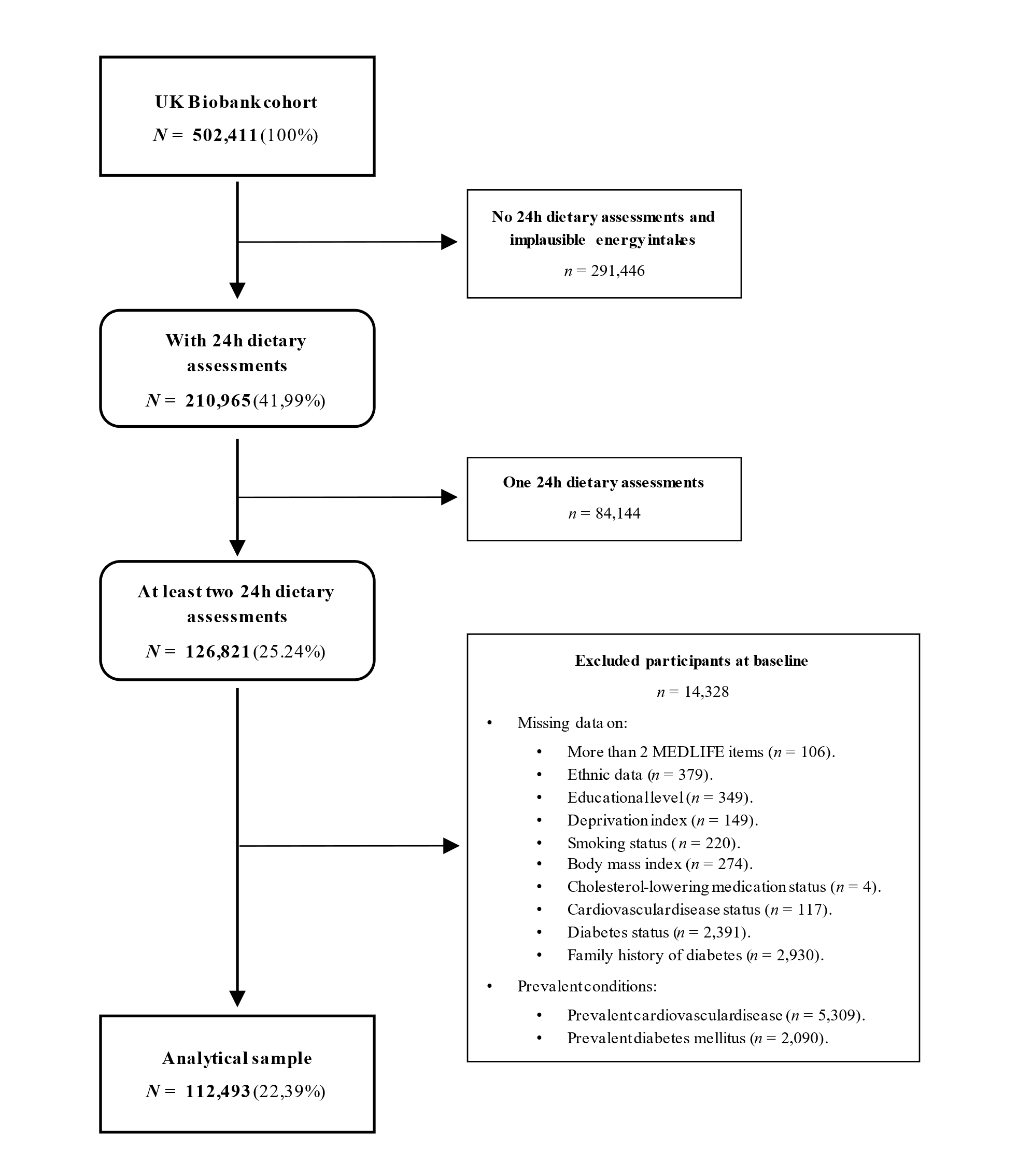


**Supplementary Figure S2.** Associations between the MEDLIFE index (per 1 point) and risk of type 2 diabetes in the UK Biobank, excluding items from the score (*N* = 112,493**)**.


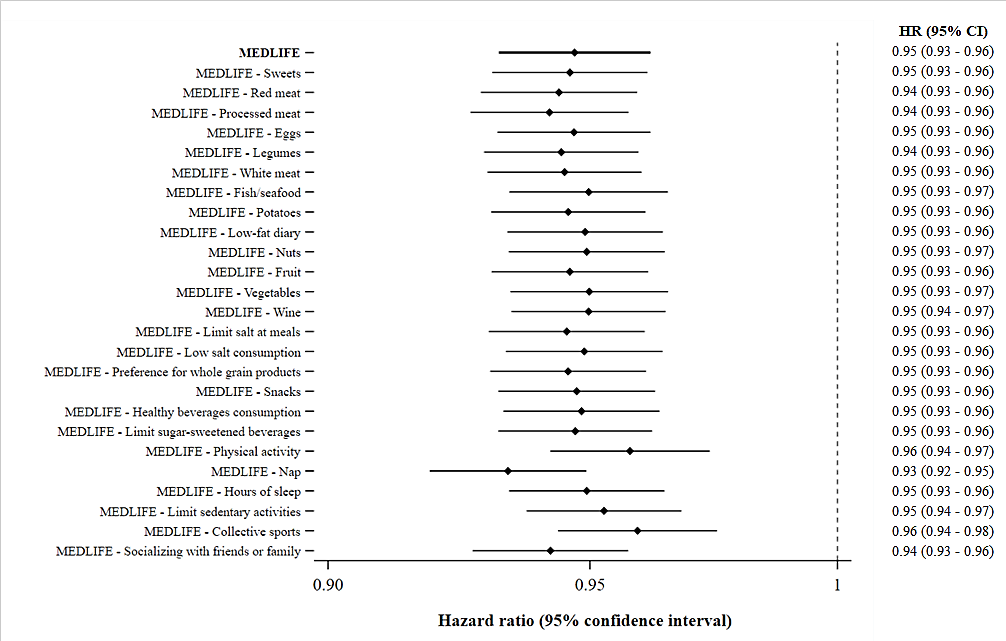


Adjusted for sex, age, ethnicity, education, deprivation index, region of assessment, smoking status, energy intake, hypertension, cholesterol-lowering medication, body mass index, family history of diabetes, and the subtracted item. Abbreviations: *N*, total number of participants.
